# Unusual origins of cardiac insufficiency: a case of iliac arteriovenous fistula post-spinal disc surgery

**DOI:** 10.1186/s13019-024-02664-4

**Published:** 2024-04-09

**Authors:** Zheng-Ji Chen, Nouman Ahmad, Ling-Jin Huang

**Affiliations:** Department of Cardiovascular Surgery, Xiangya Hospital, Central South University, No.87, Xiangya Road, Kaifu District, Changsha, Hunan 410000 China

**Keywords:** Cardiac insufficiency, Arteriovenous fistula, Imaging, Case report

## Abstract

In this case report, we present the unique and intriguing case of a 57-year-old man who experienced exertional palpitations and shortness of breath for 5 years. He was diagnosed with idiopathic heart failure three years ago, leading to diuretic treatment. Physical examination revealed notable left lower extremity swelling, severe varicose veins, and cardiac murmurs. Echocardiography showed significant cardiac enlargement and severe functional mitral and tricuspid valve regurgitation. Computed tomography (CT) imaging uncovered a 10 mm left common iliac arteriovenous fistula, causing abnormal early filling of the inferior vena cava (IVC) and marked IVC dilation. Open surgical repair of the arteriovenous fistula resulted in symptom relief and improved cardiac function. This case underscores the importance of considering unusual causes in heart failure patients and highlights the value of early diagnosis and intervention in complex cardiac-vascular interactions.

## Background

Cardiac insufficiency, characterized by structural or functional alterations in the heart, results in compromised ventricular pumping efficiency and impaired filling capacity. This condition primarily stems from various etiological factors, such as myocardial damage, cardiac overload, or insufficient ventricular preload, which can lead to decreased cardiac output [1].

In this case report, we present a unique clinical scenario where cardiac insufficiency arises as a consequence of an abnormal arteriovenous shunt. The significance of this case lies in its rarity and atypical presentation, highlighting the need for a comprehensive understanding of the diverse mechanisms contributing to cardiac insufficiency. By examining this case in detail, we aim to shed light on the intricate relationship between cardiac physiology and unexpected vascular anomalies.

In the following sections, we will provide a comprehensive overview of the patient’s clinical history, diagnostic assessment, management, and outcome, offering insights into the pathophysiological mechanisms underlying cardiac insufficiency in the context of an abnormal arteriovenous shunt.

## Case Presentation

A 57-year-old man presented with a 5-year history of exertional palpitations and shortness of breath. Three years ago, he was diagnosed with Idiopathic heart failure at a clinic and has been receiving diuretic treatment since then. Additionally, he had a surgical history of lumbar disk protrusion surgery performed 21 years ago.

Upon physical examination, the patient exhibited significant swelling in his left lower extremity, along with visible hyperpigmentation and severe varicose veins. In the precordial area, a Grade 2 early systolic murmur was detected. An unusual continuous murmur with an associated thrill was noted over the left lower abdomen.

Echocardiography revealed marked enlargement of the entire heart, with an end-diastolic diameter of 65 mm for the left ventricle and 53 mm for the right ventricle. Severe functional regurgitation was observed in both the mitral and tricuspid valve.

**Fig. 1:**
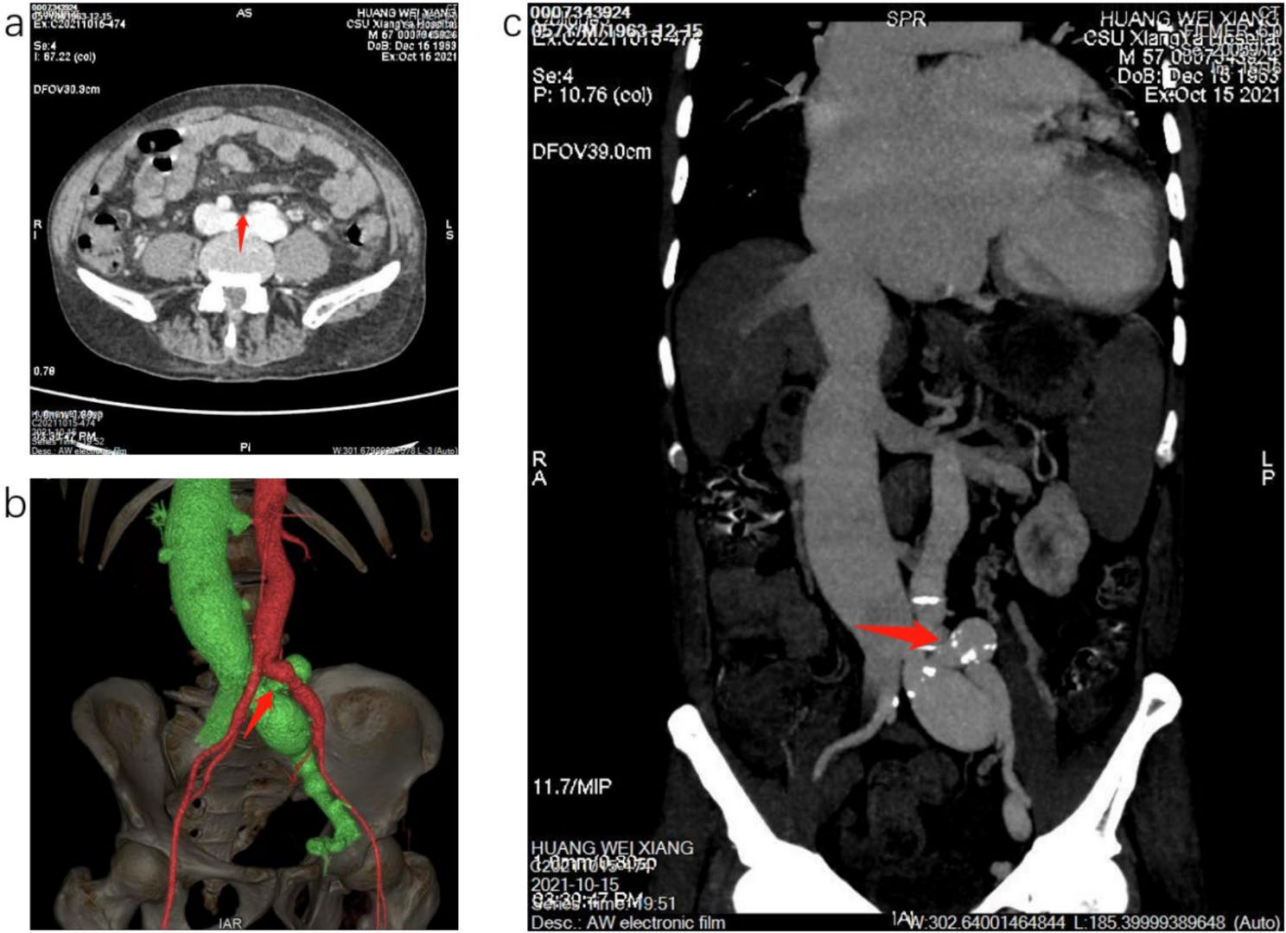
Computed tomography angiography (**a** axial, **b** coronal, and **c** 3D volume-rendering image) showing left iliac arteriovenous fistulae (marked by red arrows)

**Fig. 2:**
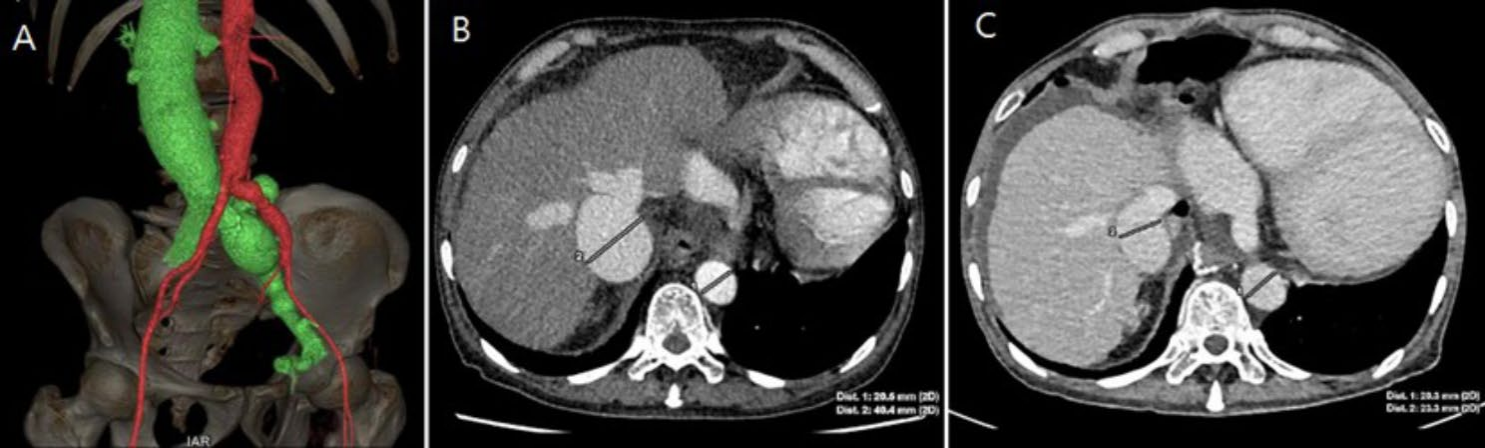
Computed tomography angiogtaphy of the inferior vena cava, left iliac vein, and right upper common iliac vein **(A)**. Inferior vena cava diameter before the surgery **(B)** and after the surgery **(C)**

Further evaluation via computed tomography (CT) scan revealed a 10 mm left common iliac arteriovenous fistula. 3D volume-rendering CT angiography showed abnormal early filling of the inferior vena cava (IVC), left iliac vein, and right upper common iliac vein (Fig. 1). The IVC appeared significantly dilated, measuring approximately twice the diameter of the descending aorta on the same plane (Fig. 2).

**Fig. 3:**
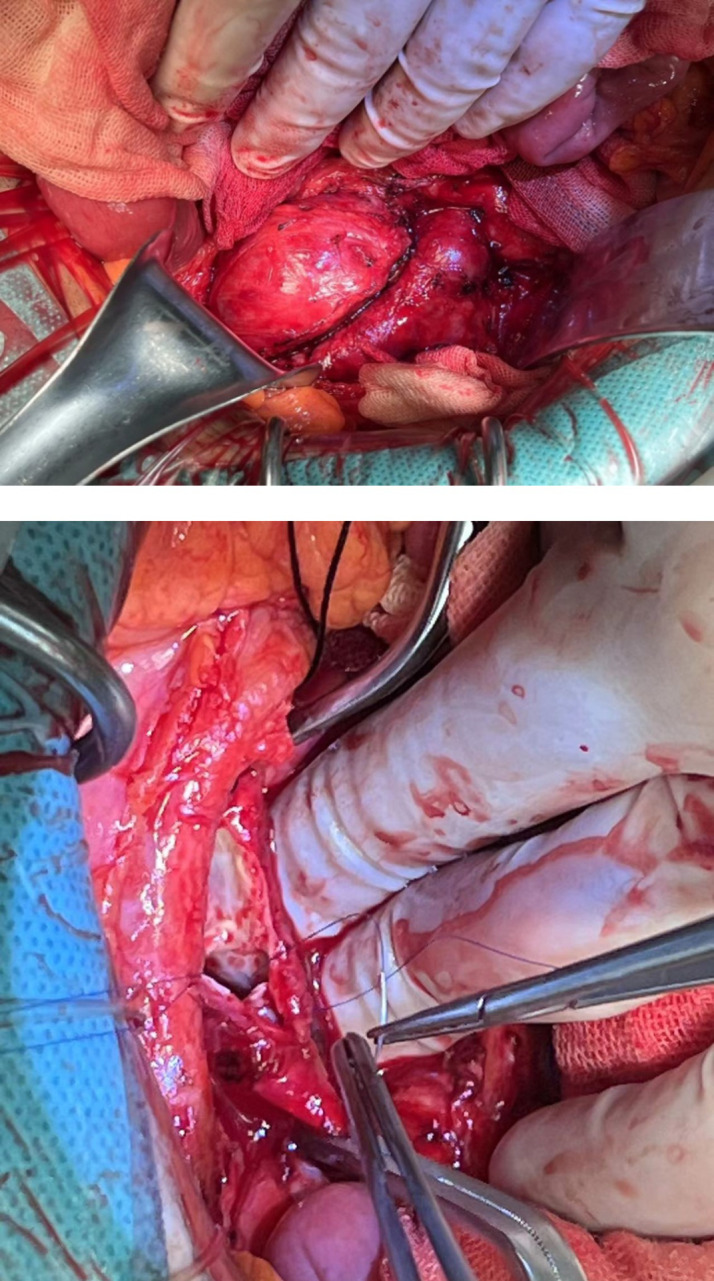
Intraoperative image showing the (**a**). iliac arteriovenous and (**b**). direct closue of the fistula with intermittent 4 − 0 prolene suture

Due to the small size of the patient’s left femoral artery, large size of fistula, which were contraindications for stent repair [2, 3], an open surgical procedure was performed through a transabdominal incision. Fistula was found between the posterior wall of the left iliac artery and iliac vein. The size was about 10 mm and the iliac vein was extremely dilated. The fistula was repaired directly with intermittent 4 − 0 prolene suture (Fig. 3). The incision of the iliac artery was closed followed by the closure of abdominal layers and the abdominal incision. The iliac vessel mumur disappeared after the surery.

**Fig. 4:**
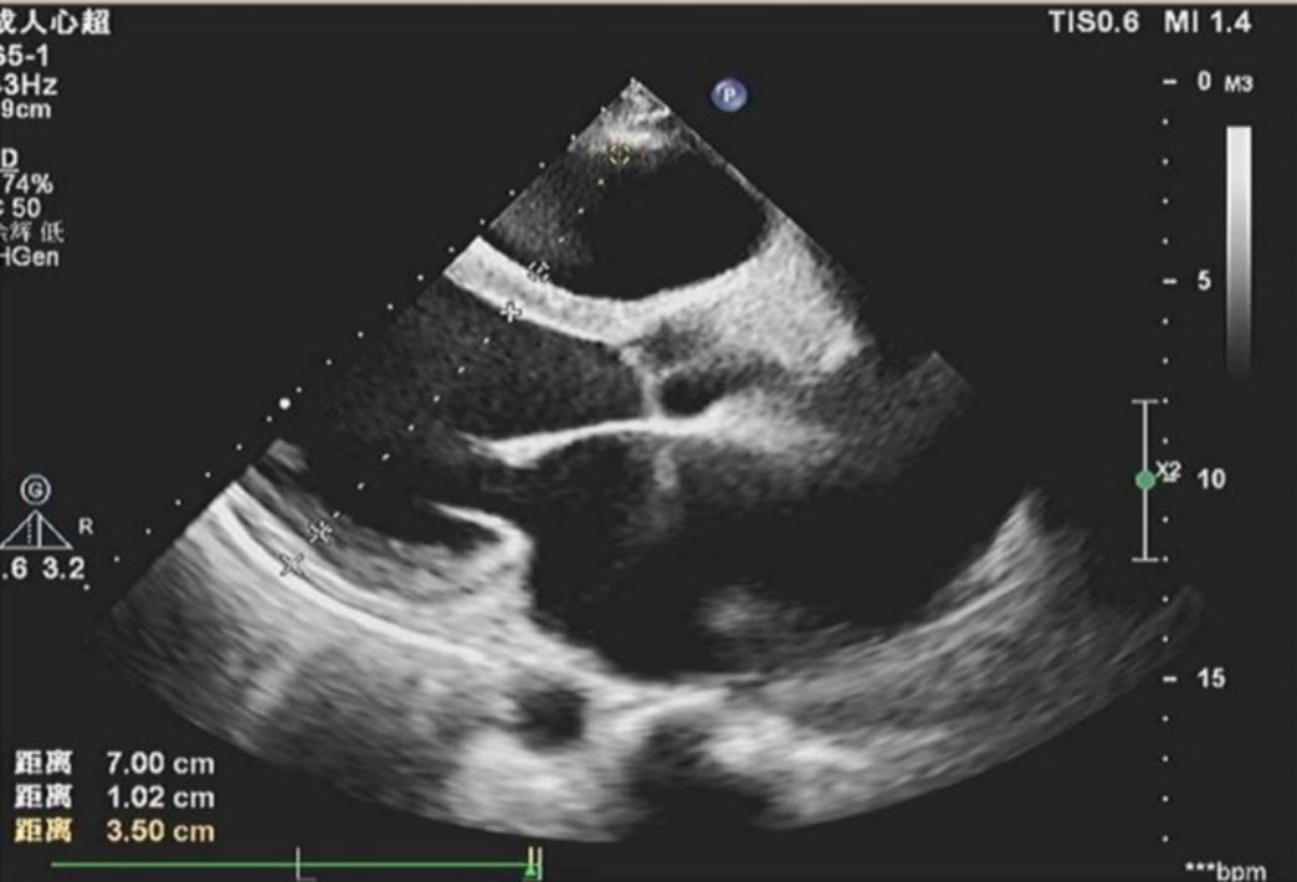
A follow up Echo showing significantly reduced functional mitral and tricuspid valve regurgitation

The patient’s post-operative recovery was favorable, with a notable improvement in symptoms and satisfaction. A follow-up echocardiography examination conducted one week after the operation demonstrated significantly reduced functional mitral and tricuspid valve regurgitation (Fig. 4). Additionally, the diameter of the inferior vena cava had returned to almost normal, measuring 23 mm in the CT scan (Fig. 2).

## Discussion

Clinically, unexplained cardiac enlargement and cardiac insufficiency present diagnostic challenges. In this case, the patient exhibited generalized cardiac enlargement and concurrent multiple valve insufficiencies, which could easily be misdiagnosed as primary cardiomyopathy. Additionally, the marked dilation of the inferior vena cava, often viewed as a secondary change in response to cardiac failure, further complicated the clinical picture.

What sets this case apart is the extreme extent of inferior vena cava dilation, far surpassing what is typically observed in cases of heart failure alone. Coupled with the patient’s history of lumbar spine disc surgery, the discovery of an iliac arteriovenous fistula emerged as a significant revelation. Such fistulas are exceedingly rare complications of spinal disc surgery [4]. This may be caused by the use of instruments such as the pituitary rongeur or forceps to perforate the anterior spinal ligament, which is anatomically very close to the vessels. Or it could be retroperitoneal inflammatory processes leading to adhesion of the vessel and the disc [5]. The massive iliac arteriovenous shunt imposed a substantial volume load on the heart, resulting in cardiac enlargement and insufficiency. Notably, congestive heart failure typically develops rapidly in such cases. However, this patient’s prolonged history of symptoms was exceptional.

Early detection and intervention to correct the fistula proved instrumental in halting the progression of heart enlargement and cardiac insufficiency. This case serves as a poignant reminder of the critical importance of considering uncommon etiologies in patients with cardiac insufficiency and highlights the value of swift diagnosis and treatment.

## Conclusion

In summary, this case report illuminates the complexities of diagnosing cardiac enlargement and insufficiency, emphasizing the need to explore beyond conventional explanations, especially when faced with protracted and enigmatic clinical presentations. The identification of an iliac arteriovenous fistula related to lumbar spine disc surgery offers a valuable lesson in recognizing rare complications. Prompt intervention in cases such as these can halt the progression of heart enlargement and cardiac insufficiency, underscoring the importance of early detection and management to optimize patient outcomes.

## Data Availability

All data generated or analysed during this study are included in this published article.
